# High-altitude wind resources in the Middle East

**DOI:** 10.1038/s41598-017-10130-6

**Published:** 2017-08-29

**Authors:** Chak Man Andrew Yip, Udaya Bhaskar Gunturu, Georgiy L. Stenchikov

**Affiliations:** 1 King Abdullah University of Science and Technology, Applied Mathematics and Computational Science, Thuwal, 23955-6900 Saudi Arabia; 2 King Abdullah University of Science and Technology, Earth Science and Engineering, Thuwal, 23955-6900 Saudi Arabia

## Abstract

In the Middle East, near-surface wind resources are intermittent. However, high-altitude wind resources are abundant, persistent, and readily available and may provide alternative energy resources in this fossil-fuel-dependent region. Using wind field data from the Modern-Era Retrospective Analysis for Research and Applications Version 2 (MERRA-2), this study identifies areas favorable to the deployment of airborne wind energy (AWE) systems in the Middle East and computes the optimal heights at which such systems would best operate. AWE potential is estimated using realistic AWE system specifications and assumptions about deployment scenarios and is compared with the near-surface wind generation potential with respect to diurnal and seasonal variability. The results show the potential utility of AWE in areas in the Middle East where the energy demand is high. In particular, Oman and Saudi Arabia have a high level of the potential power generation with low annual variability.

## Introduction

In 2015, with a record 63 GW added to the total global renewable power capacity of about 433 GW in 2014, renewable energy became the largest contributor to new power-generating capacity in the United States and Europe and the second largest contributor in China^1^. Near-surface wind power is a mature technology and a fast-growing renewable power source that has the potential to contribute substantially to the reduction of greenhouse gas emissions^2^. In the Middle East, wind power as a renewable energy resource has attracted interest primarily because of potential economic savings and energy resource diversification^3^. Economic savings from wind power in the region would be primarily realized through the opportunity costs from saving fossil fuels from use in domestic power generation. With round-the-clock availability, wind power also provides a way to diversify the energy mix in the oil-exporting countries of the Middle East. Moreover, the renewable energy sector creates jobs that require technical expertise that is currently being cultivated in the younger generations of the local workforce, leading to a sustainable and knowledge-based economy^3^. These factors, coupled with the recent drop in oil prices, have incentivized wind power harvesting in the Middle East.

A recent survey of near-surface wind resources showed that there are some areas with high and persistent winds in the Middle East^4^. However, near-surface wind resources in the Middle East are not as abundant or persistent as are wind resources in other regions of the world^5, 6^. Generation of airborne wind energy (AWE) is possible in areas with little near-surface wind^7^. Indeed, higher and steadier power generation from AWE can be attained due to the greater availability of persistent wind resources at higher altitudes^7^. AWE is generated using airborne devices that are connected to a ground station by a tether. Instead of land-based wind turbines that operate near the surface, AWE uses devices that extract kinetic energy from the wind available above the surface layer of the atmosphere and turn it into electricity^8^. There are currently two major types of AWE generators: drag type devices with generators on board with a tether that transmits electrical power, and lift type devices that transmit mechanical power in reeling the tether connected to a ground based generator. Active projects include KiteGen (drag) and Makani (lift), both are under active development and pilot studies. KiteGen is in the development of a 3 MW ground-based device and Makani is working on a 600 kW on-board generator prototype. Details of the technologies and their working principles have been described in prior studies^9^.

There have been interests in AWE in various geographical regions, most recently in Northern Ireland^10^. The potential of AWE in the Middle East has not previously been explored. This study contributes to the evaluation of AWE in the Middle East by identifying areas favorable for AWE system (AWES) deployment; analyzing the diurnal and seasonal variability of airborne wind resources; determining a range of optimal altitudes for AWES deployment; and estimating AWE generation potential compared with near-surface wind generation potential. The results reported in this article represent a survey of high-altitude wind resources at 3-hourly temporal resolution. Further downscaling studies and measurement campaigns, which are beyond the scope of this article should be conducted on site for siting purposes.

## Results

The analysis of AWE in the Middle East begins by studying the vertical characteristics of horizontal wind in the boundary layer relevant to AWE. Horizontal wind speed data at 13 pressure levels are gathered from the Modern-Era Retrospective Analysis for Research and Applications Version 2 (MERRA-2)^11^. Wind speed maxima (WSM), the maximum wind speed at each time step across pressure levels at each grid cell, is used to measure the magnitude and altitude of winds at high-altitude. The average magnitude and variability of WSM were computed to summarize the vertical profile of WSM at every grid cell in the domain of interest by the time of day and season. Variability in the magnitudes of WSM and the altitudes at which they occur (H) is also characterized to gain an overview of the vertical range of operations and relative ease of deployment of AWE systems across the spatial domain where the length and the consequent weight of the tether create a deployment constraint. The following plots with discrete color scales are generated using the k-means algorithm, which clusters values around centroids that become the labels of each interval on the color scale.

### Average WSM

The boundary layer over the Arabian Peninsula could reach up to 6 km above the surface in summer^12^. The wind speed in the lowest 3 km of the boundary layer does not necessarily increase monotonically with altitude due to the presence of low-level jets (LLJs)^7^. LLJs are streams of fast-moving air with super-geostrophic speeds located over 100 m above ground and formed by various mechanisms, such as inertial oscillations^13^ and baroclinicity over the sloping terrain due to the horizontal temperature gradient^14^. Both mechanisms are discussed in the seminal book^15^.

**Figure 1:**
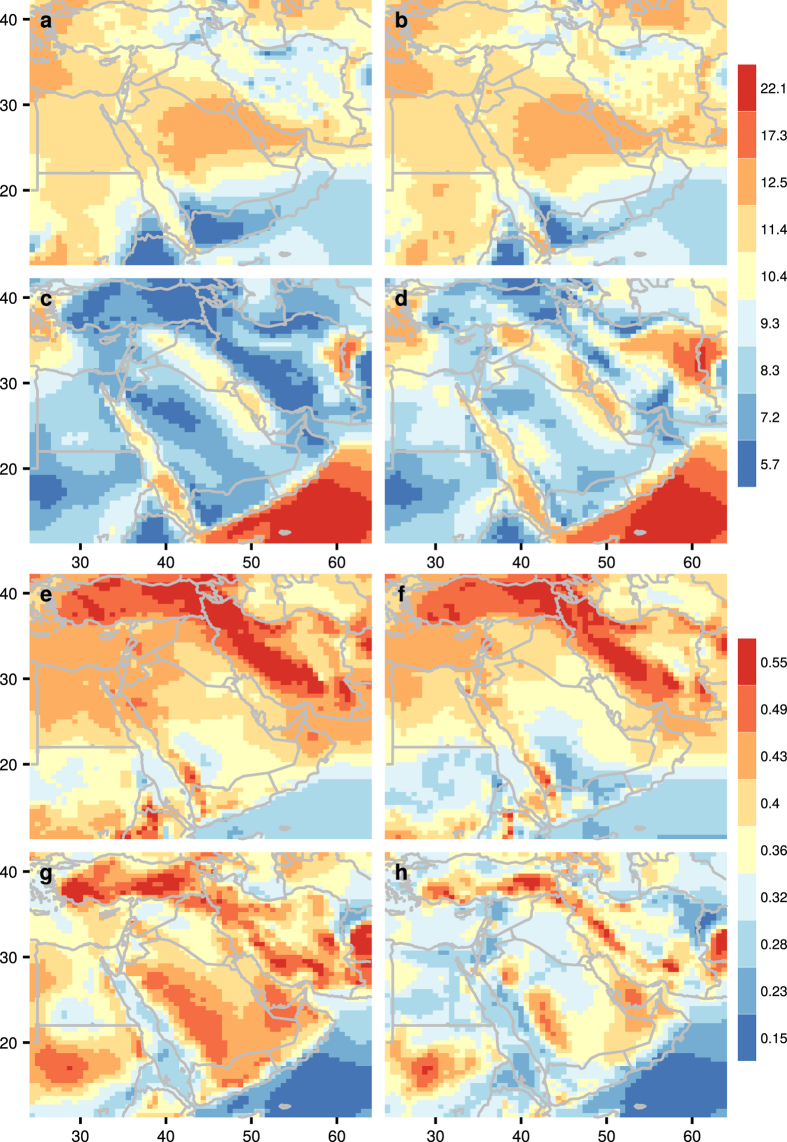
Average wind speed (m/s) of WSM during: (**a**) day time in January, (**b**) night time in January, (**c**) day time in July, (**d**) night time in July. CV in the wind speed of WSM during: (**e**) day time in January, (**f**) night time in January, (**g**) day time in July, (**h**) night time in July. The figure including the map and all the text elements has been plotted using the software R version 3.3.2.12 Panels (a–d) share a uniform color scale from 5 to 13 m/s; values larger or smaller than the boundaries share the bounding colors. These boundaries are informed by the wind speed distributions (Fig. S1) and the wind speed maxima vertical profiles (profiles at selected locations are illustrated in Fig. S2).

Figure 1(a–d, with individual color scales due to differences in wind speed distribution) shows that there are areas with average WSM ($\overline{WSM}$) over 12 m/s over eastern Saudi Arabia, Kuwait, Bahrain, and Qatar in winter. In summer, areas with persistent winds are located along the coast of Yemen and Oman, due to the presence of the Somali Jet associated with the Indian summer monsoon. A high AWE resource is located north of 22°N in winter. South of 22°N, high AWE resources appear to be in coastal areas.

$\overline{WSM}$ is much higher over the Arabian Sea during the summer than during the winter. A slightly higher $\overline{WSM}$ is observed in summer in western Iran. This WSM is more prominent during the night than during the day. Higher $\overline{WSM}$ is observed during the winter in most areas in the region, particularly in central and northwestern Saudi Arabia, across the Gulf of Oman, and in western Turkey. $\overline{WSM}$ experiences little diurnal variation, except in July, when $\overline{WSM}$ is higher at night in western Saudi Arabia, Iran, and parts of Turkey.

### Variability in WSM

Variability in WSM (CV_WSM_) is measured by the coefficient of variation (CV), a dimensionless measure of spread that describes the variability relative to the mean. Figure 1(e–h) shows that CV_WSM_ decreases by an average of 0.1 during the night in comparison with during the day in both January and July. Larger decreases above 0.1 are observed along the northern coast of the Red Sea and near the mountain ranges in Yemen. In July, a decrease of over 0.1 in variability during the night is also seen along the Mediterranean coast of North Africa and the Middle East and in Eastern Iran. The daytime WSM has higher variability in the southern coast of the Black Sea in January than in July.

CV_WSM_ is lower by more than 0.1 in summer than in winter along the west coast of the Red Sea, the southern coast of the Arabian Peninsula, and the land area north of 20° N. Over Sudan, central and eastern Saudi Arabia, and the Arabian Gulf, CV_WSM_ is lower by around 0.1 in winter than in summer.

In summer, CV_WSM_ is higher during the night than during the day over most land areas except over Sudan and the Gulf of Aden. The spatial variation of CV_WSM_ is minimal except for areas surrounding the Gulf of Aden; for instance, the CV_WSM_ in the daytime in winter is lower than that during the night. There is also slightly higher variation in CV_WSM_ along the Arabian coast of the southern Red Sea during the day.

### Altitude of WSM

**Figure 2:**
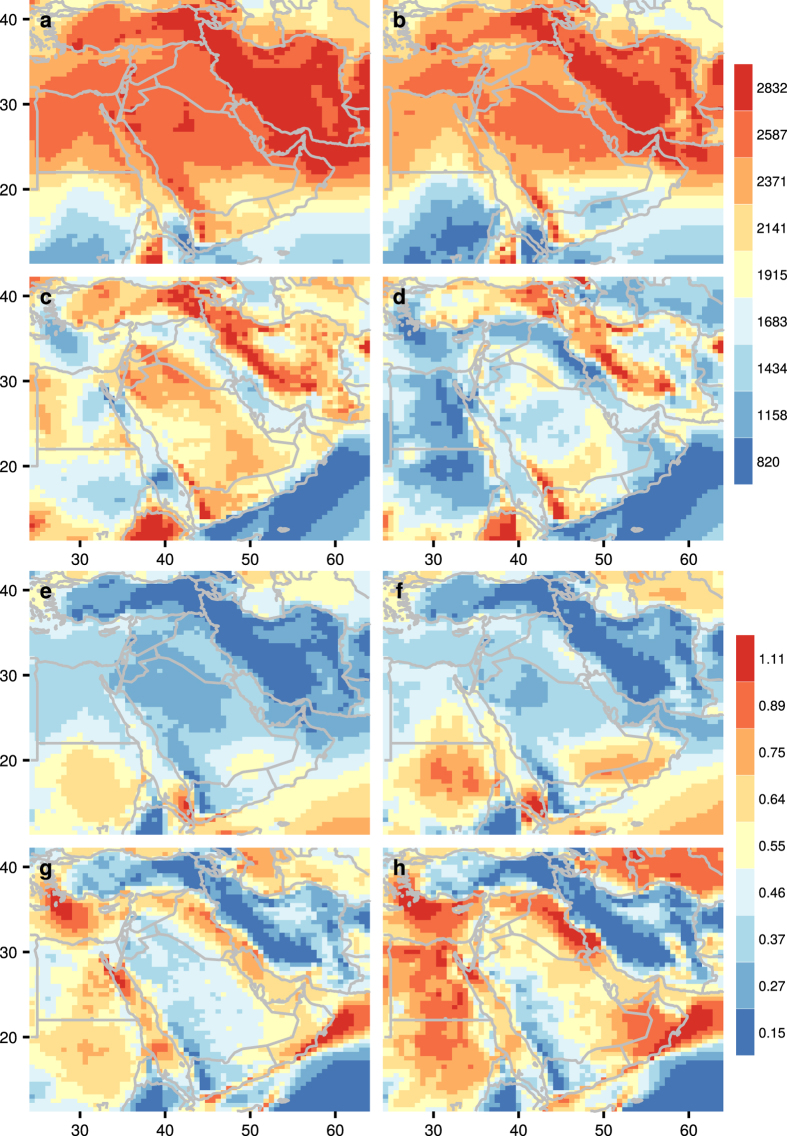
Average altitude (meters above sea level, mASL) of WSM ($\overline{H}$) during: (**a**) day time in January, (**b**) night time in January, (**c**) day time in July, (**d**) night time in July. CV in the altitude of WSM (H) during: (**e**) day time in January, (**f**) night time in January, (**g**) day time in July, (**h**) night time in July. The figure including the map and all the text elements has been plotted using the software R version 3.3.2^22^.

The average altitude of WSM ($\overline{H}$) (Fig. 2a–d) follows similar diurnal patterns in January. In July, WSM is at a relatively low altitude over the Mediterranean coast of the Middle East at about 1 km. Also, a reduction in $\overline{H}$ (over 500 m) occurs over Egypt, northwestern Saudi Arabia, and northern Oman in July during the night when compared with the day.

WSM occurs at a much lower altitude during the night in summer than during the night in winter, particularly along coastal areas where the reduction in $\overline{H}$ is more than 500 m. Egypt experiences a reduction of over 1 km in $\overline{H}$ at night in winter compared with at night in summer. Exceptions are found where the Red Sea joins the Gulf of Aden with an increase of 500 m in $\overline{H}$.

### Variability in WSM altitudes

Variability in WSM altitudes (CV_H_) is measured by CV. Low CV_H_ (Fig. 2) is associated with areas where $\overline{H}$ is high. High CV_H_ is associated almost exclusively with the Mediterranean coast of the Middle East, the southern coast of Oman, and Egypt, where the average altitude is below 1 km.

Seasonal variation in CV_H_ is consistent during the day and night. Areas above 20° N experience a higher CV_H_ in summer than in winter. Areas below 20° N experience a higher CV_H_ of more than 0.5 in winter than in summer. Central Saudi Arabia, northern Iran, western Turkey, Yemen, Sudan, and Ethiopia experience little variation in CV_H_ in summer and winter.

In summer, Egypt, northern and eastern Saudi Arabia, Oman, the Mediterranean coast of the Middle East, and Iraq experience slightly higher CV_H_ of more than 0.2 during the night than during the day. In winter, the same is observed for eastern Sudan and Oman, especially along the coast.

### Regional AWE potential

We explored regional AWE potential in three ways:

- Examining the average capacity factor (CF) of AWE generation in the region and identifying areas of interest, where CF is the ratio of power generation to the nameplate (maximum) capacity of the AWE system.
- Computing annual energy production at the country level given certain assumptions about AWES deployment.
- Comparing AWE potential with near-surface wind energy potential.

#### Average power generation at WSM

**Figure 3:**
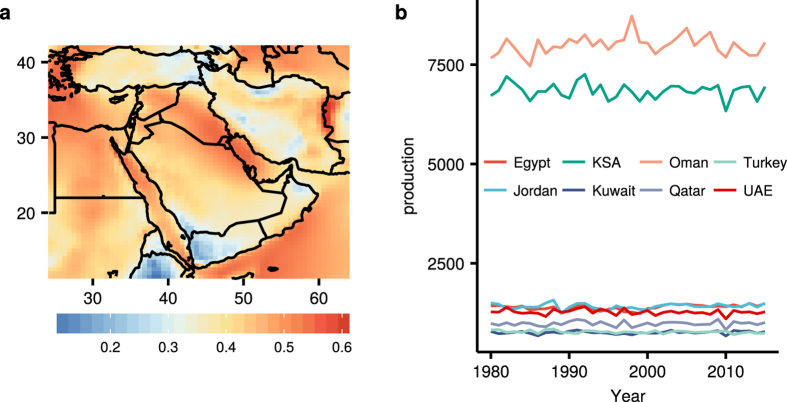
Map of AWE potential. The CF of the 3 MW AWE system (left) and annual AWE generation per capita (kWh) by country (right). The figure including the map and all the text elements has been plotted using the software R version 3.3.2^22^.

A map of AWE feasibility (Fig. 3a) is constructed using the CF computed from the power curve of a ref. 3 MW AWES17. Among the areas with CF greater than 0.5 are Kuwait, Syria, Jordan, and Egypt. The Aegean coast of Turkey is another preferable location for deployment. The eastern border of Iran also has exceptional potential with a favorable terrain, where there are contiguous open areas. The southern coasts of Yemen and Oman have similar terrain and a high CF. AWE potential over Saudi Arabia is the most abundant in the northeast on the border with Kuwait and along a thin coastal strip along the northern Red Sea.

#### Annual per capita energy production at WSM

Figure 3b shows the projected average annual AWE generation per capita for each country. The average annual generation per capita in Oman and Saudi Arabia stands out for more than triple the per capita generation of other countries in the region mostly due to the high wind speed (Oman) and the vast area (Saudi Arabia). The coefficient of variation in annual generation per capita is highest (0.06) among Qatar, Bahrain, and Palestine, and lowest (0.02) among Yemen, Oman, and Saudi Arabia. The high average annual generation and low annual variability make Oman and Saudi Arabia promising candidates for large-scale AWE deployment.

Table 1 presents the AWES potential for each country and the total potential power generation and energy consumption per capita for 2010. The number of deployable generators ranges from one to over fifteen thousand, reflecting the vast differences in the available land across the selected countries in the region. The spatial density of AWE systems varies from 0.18 to 0.96 per 100 km^2^, reflecting the different levels of urbanization and terrain features in the region. Oman, Saudi Arabia, Iraq, Egypt, and Yemen have power generation potential from AWE that could meet over 75% of the energy consumption required for 2010 at the assumed deployment level.

**Table 1:** Consumption (C) and potential AWE generation (G) for countries in the Middle East (per capita) with the maximum number of deployable generators (MNG), density of generators per 100 km^2^ (GD), and the population (P).

Country	MNG	GD (/100 km^2^)	P (million)	C (kWh)	G (kWh)
Oman	2836	0.91	3.42	5991	7841
Saudi Arabia	18392	0.96	28.69	8022	6463
Iran	12587	0.78	66.43	2634	1811
Iraq	3618	0.83	31.13	1187	1473
Jordan	780	0.88	6.34	2216	1462
Egypt	9644	0.96	83.08	1670	1437
Yemen	3491	0.77	23.82	259	1254
UAE	602	0.85	4.80	10746	1123
Syria	1497	0.81	20.18	1809	938
Qatar	63	0.57	0.83	15075	845
Turkey	6215	0.80	76.81	2498	812
Kuwait	145	0.84	2.69	16759	683
Cyprus	22	0.38	0.53	4622	488
Israel	184	0.83	7.23	6953	313
Lebanon	42	0.42	4.02	3475	122
Palestine	37	0.58	4.12	—	112
Bahrain	1	0.18	0.73	17578	22

C and G data are for 2010 from IEA Statistics© OECD/IEA 2014.

### Comparison with near-surface wind resources

**Figure 4:**
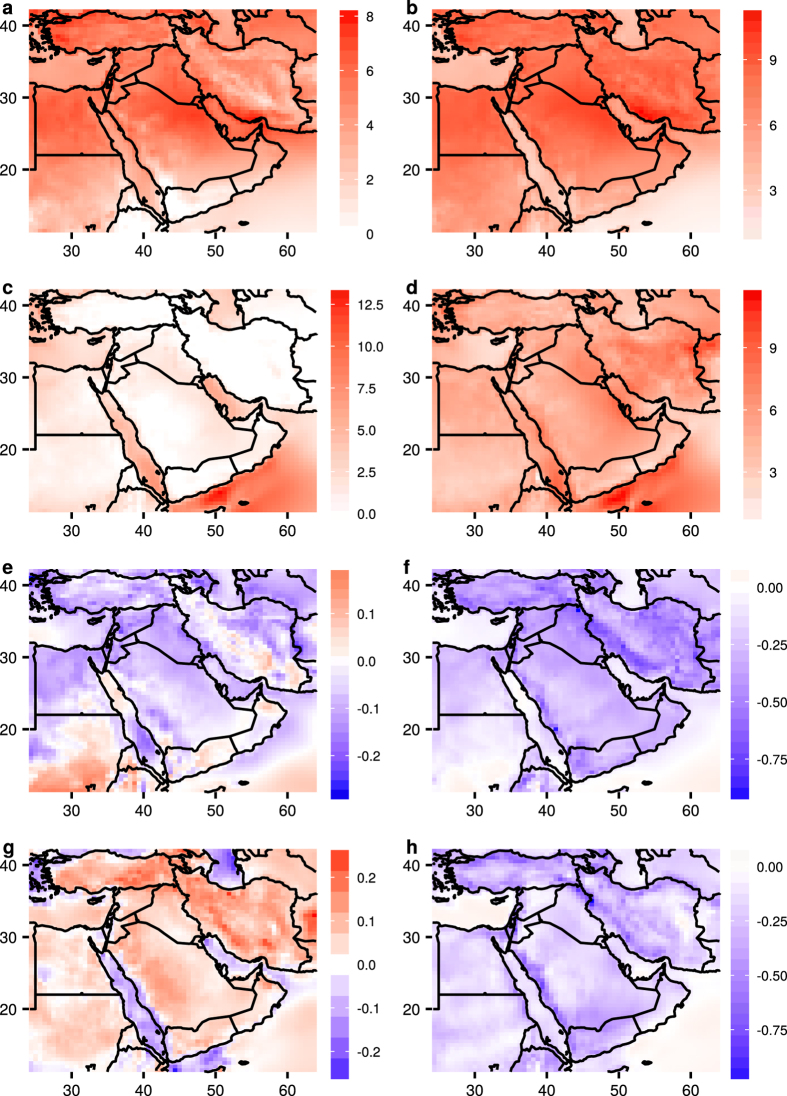
Difference in the average wind speed (m/s) between WSM and WS at 80 m during: (**a**) day time in January, (**b**) night time in January, (**c**) day time in July, (**d**) night time in July. The difference in CV in wind speed between WSM and WS at 80 m during: (**e**) day time in January, (**f**) night time in January, (**g**) day time in July, (**h**) night time in July. The figure including the map and all the text elements has been plotted using the software R version 3.3.2^22^.

In Fig. 4(a–d), the average wind speed at WSM is compared with the average wind speed (WS) at 80 m by season and time of day to understand the advantages of AWE. The advantages are most obvious in winter when jet streams are active. In summer, AWE appears to be more advantageous near the coast of the Red Sea, the Arabian Gulf, and the Gulf of Aden. The gain of AWE over near-surface wind resources is minimal in regions with a more complex terrain, such as in western Saudi Arabia and Yemen, especially during the day. In summer daytime, surface heating overland causes a well-mixed and 5-km deep boundary layer. The strong turbulent mixing of momentum equalizes winds at different altitudes^12^.

The difference in the CV (Fig. 4e–h) shows that WSM is less variable than the near-surface wind in winter. In summer, the variability increases slightly over land during the day except in coastal areas. At night, coastal areas experience a large reduction in variability in WSM compared with that of near-surface wind, likely due to the prominence of low-level jets.

## Discussion

In this study, areas favorable for AWES deployment in the Middle East are identified with the diurnal and seasonal characteristics of AWE potential characterized. The abundance of wind resources increases with altitude, except in the coastal areas in summer nights. Variability in wind resources increases with altitude over coastal areas in summer but not so much in winter. Optimal altitudes of AWES deployment were computed in light of current technological constraint on the length of the tether. The regional AWE potential was estimated using a realistic power curve specification with assumptions about deployment conditions and spatial distribution. Average WSM is higher over land in general in winter north of 20° N and over Sudan. Coastal WSM is more prominent in summer. There is a lower variability in coastal areas in summer. The average altitude of WSM is lower during the summer, especially in coastal areas where the variability is higher. The per capita annual energy generation demonstrates the potential of AWES in fulfilling electricity needs at current levels for several countries in the Middle East. In particular, Oman and Saudi Arabia have a high level of the potential power generation with low annual variability. Our estimates also compare favorably to the near-surface wind power potentials from a previous study in which higher average wind speeds and less variability were observed^4^. This finding hints at the potential utility of AWE for areas with high energy demand in the region.

In future studies, we will focus on characterizing the regional impact of AWES, where annual production estimates across countries in the region would offer insight into the potential contribution of AWE to respective electricity grids. The effects of spatial aggregation of AWES in reducing power variability would offer insights into the impacts of large-scale deployment of AWES on the grid. The untapped potential and technical viability of AWES suggest that studies on spatial optimization of wind power systems where conventional turbines and AWES may co-exist are warranted. AWES presents an excellent opportunity to champion the technological transfer and development of a maturing next-generation technology in a region with an increasingly knowledge-based and energy-intensive economy.

## Methods

Wind speed vertical profiles were constructed using the three-hourly instantaneous output from MERRA-2, a project from the US National Aeronautics and Space Administration (NASA) on a 0.5° (latitude) × 0.625° (longitude) grid^11^. The horizontal wind vectors (*U*, *V*) and altitudes (*H*) were retrieved at 13 pressure levels from sea-level to 700 hPa. The data spanned 35 years from 1 January 1980 to 31 December 2015. Wind speed (*u*) was computed using $u=\sqrt{{U}^{2}+{V}^{2}}$. The long record-length, even spatial and temporal coverage, state-of-the-art spatial and temporal resolutions as compared to other reanalyses products and prior validations in various studies make MERRA-2 a valuable dataset for wind resource assessment in the area of interest where reliable long-term measurements are rare. MERRA-2 assimilates conventional observations such as radiosondes and surface station observations from various sources, with over 1200 surface stations and 150 radiosonde locations in the region of interest^16, 17^.

**Figure 5:**
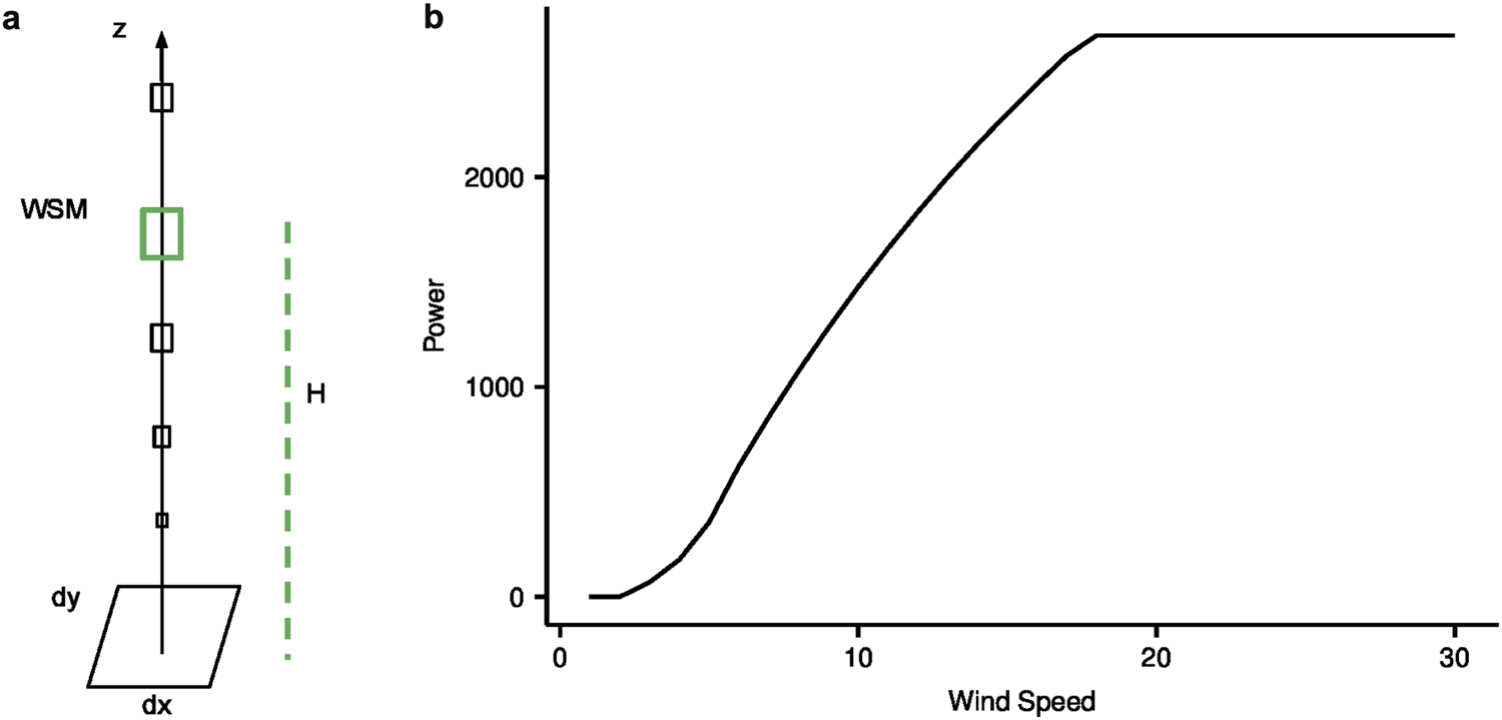
(**a**) Definition of wind speed maxima (WSM) and its associated altitude (H), where the size of the box indicates the magnitude of the wind speed (**b**) Power curve, power (kW) as a function of wind speed (m/s), of a lift-type groud-based AWES of 3 MW rating. The figure and all the text elements in (**b**) has been plotted using the software R version 3.3.2.

WSM was defined as the maximum wind speed in the lowest 13 pressure levels from the sea surface to 700 hPa. The associated altitude at which WSM occurs is H (Fig. 5a). Wind resource at high-altitude was characterized from WSM and H. The altitude of these pressure levels from the ground varied with the earth’s topography. Engineering constraints on the tethers due to increasing weight with length limited our focus to the first 3 km of the boundary layer^18^. The boundary layer in the Arabian Peninsula varies in thickness and could reach 5 km in height in the afternoon^19^. A similar definition of WSM was previously used with an imposed lower bound of 10 m/s^18, 20^. However, maximum wind speed in this study includes winds below 10 m/s because it has been technically demonstrated that current AWES could operate at nameplate capacity with wind speeds under 10 m/s^21^. For this study, the cut-in speed of the device was 2 m/s and the wind speed at which the nameplate capacity was reached was 6 m/s^21^.

### WSM

For each grid cell in the domain, the average WSM ($\overline{WSM}$) was computed using the maximum hourly wind speed field across pressure levels over the data collection period, as follows:

1 $$\overline{WSM}=\frac{1}{n}\sum _{t=1}^{n}WS{M}_{{\rm{t}}},$$

where *WSM*_t_ = *max*(*u*_*i*_), *i* ∈ [1,13] at each hour (*t*) and *n* is the record length. Variability in WSM was characterized with the coefficient of variation (CV) for each cell as follows:

2 $${{\rm{CV}}}_{{\rm{WSM}}}=\frac{{\sigma }_{WSM}}{\overline{WSM}},$$

where *σ*_*WSM*_ is the standard deviation over time for each cell. The CV provides a dimensionless measure of variability across the spatial domain. A high CV indicates highly variable wind resource, which would be less desirable for AWES operation.

### Altitudes of WSM

The average altitude of WSM ($\overline{H}$) for each cell was calculated as follows:

3 $$\overline{H}=\frac{1}{n}\sum _{t\mathrm{=1}}^{n}{H}_{{\rm{t}}},$$

where *H*_t_ = *H*_*j*_ such that *u*_*j*_ = *max*(*u*_*i*_), *i* ∈ [1,13]. Variability in H was characterized with CV for each cell as follows:

4 $${{\rm{CV}}}_{{\rm{H}}}=\frac{{\sigma }_{H}}{\overline{H}},$$

where *σ*_*H*_ is the standard deviation over time for each cell. A high CV in H indicates the need for frequent flight adjustments of the AWES, which would be less desirable for AWES operation.

### Capacity factor

The wind power potential of AWES operating at optimal altitudes is summarized using the capacity factor of a lift-type AWES based on its power curve (Fig. 5b). The power curve is that of a prototype AWES at a test facility^21^. A lift-type AWES generates power from traction through cycles of reeling and unreeling of a tether connected between a kite and a base station. An automatically controlled airfoil in the form of a kite with flexible wings glides in the air-space above the base station. The CF was estimated based on the construction of a realistic power curve for the device. The power curve gives the power generation of a single AWES as a function of the ambient wind speed. The power curve of the simulated device has three power-generating regimes that correspond to three flight conditions of the AWES. When the wind speed is above the cut-in speed (2 m/s) but not high enough to exert the maximum force, a small amount of power is generated as the tether reels out. When the wind speed is sufficiently high to exert the maximum force (6 m/s), the tether reels out to maintain that maximum force exerted. The power generated in this second regime is proportional to the reel-out speed of the tether. When the wind speed is over the maximum possible for the device (16 m/s), the device will navigate such that the tether is at an angle from the prevailing wind direction, maintaining the maximum power generation. In this third regime, the power generation is constant at its maximum. Since the duration of one cycle of reeling for a lift-type AWES is in minutes, the device is assumed to adjust its altitude instantaneously to reach the wind speed maximum of the hour. The nameplate capacity of this prototype AWES is 3 MW.

### Deployment assumptions

The annual potential for energy production at WSM by country in the region was computed. The annual AWE generation for each country was estimated based on assumptions about system availability, system density, and spatial exclusion:

- System availability: availability of an AWES when it is not under maintenance. The study assumes a 98 % system availability with current turbine technologies, with maintenance time distributed evenly under average generation conditions.
- System density: calculated based on how many AWE systems can be deployed in an area. Assuming that a fully extended tether to reach any altitude within the chosen portion of the boundary layer is 3 km, one unit of AWES is allowed in each 10 km × 10 km grid cell.
- Spatial exclusion: areas that are excluded for AWES deployment. Terrain slopes were computed with elevation data at 15 arcsecond spatial resolution with eight neighboring points. Regions with a slope greater than 0.2 were excluded. Inaccessible terrain (wetlands, shrublands, and forests) and urban land-use were also excluded. It is further assumed that AWE systems are uniformly distributed on the available land after these exclusions.

### Data availability

The datasets analyzed during the current study are available in the repository at the Modeling and Assimilation Data and Information Services Center of NASA, https://disc.gsfc.nasa.gov/uui/datasets/M2I3NPASM_V5.12.4/summary.

## Electronic supplementary material

Supplementary Info
