# Author Correction: High-altitude wind resources in the Middle East

**DOI:** 10.1038/s41598-018-35599-7

**Published:** 2018-11-14

**Authors:** Chak Man Andrew Yip, Udaya Bhaskar Gunturu, Georgiy L. Stenchikov

**Affiliations:** 1 King Abdullah University of Science and Technology, Applied Mathematics and Computational Science, Thuwal, 23955-6900 Saudi Arabia; 2 King Abdullah University of Science and Technology, Earth Science and Engineering, Thuwal, 23955-6900 Saudi Arabia

Correction to: *Scientific Reports* 10.1038/s41598-017-10130-6, published online 29 August 2017

This Article contains an error in the Introduction where,

“Active projects include KiteGen (drag) and Makani (lift), both are under active development and pilot studies.”

should read:

“Active projects include KiteGen (ground-based generator system) and Makani (on-board generator system), both are under active development and pilot studies.”

