# Supplementary material for: High-altitude wind resources in the Middle East
Source: Sci Rep. 2017 Aug 29;7:9885. doi: 10.1038/s41598-017-10130-6 (PMC5575235; doi:10.1038/s41598-017-10130-6)
Supplement: Supplementary file 1 — Supplementary Info [file 41598_2017_10130_MOESM1_ESM.pdf]

High-altitude wind resources in the Middle East: Supplementary Information

CMA Yip, UB Gunturu, GL Stenchikov

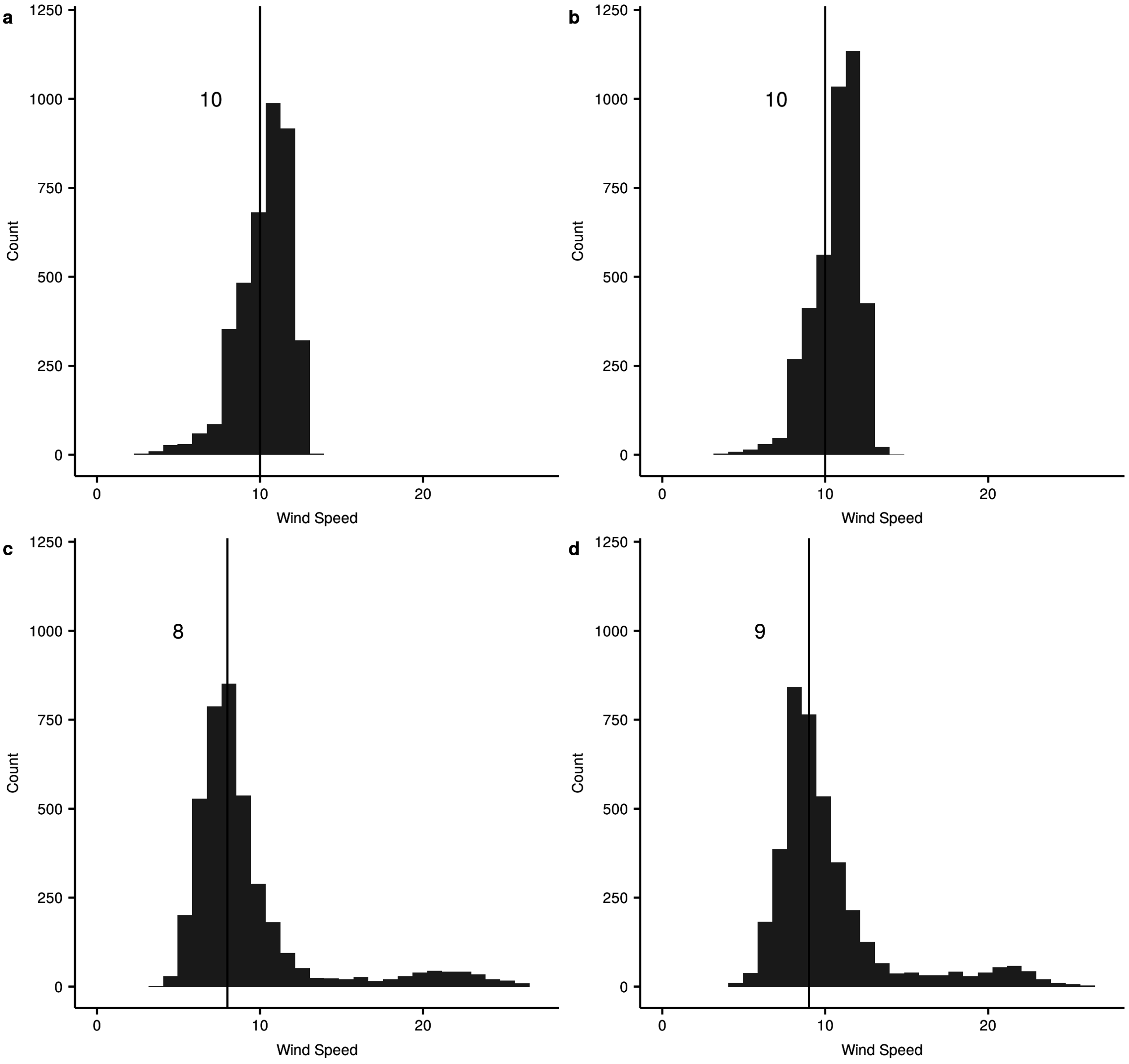

Figure S1: Histogram of the distribution of average wind speed (m/s) of WSM with the median displayed during: (a) the day time in January, (b) the night time in January, (c) the day time in July, (d) the night time in July. Note the shift of the medians across seasons.

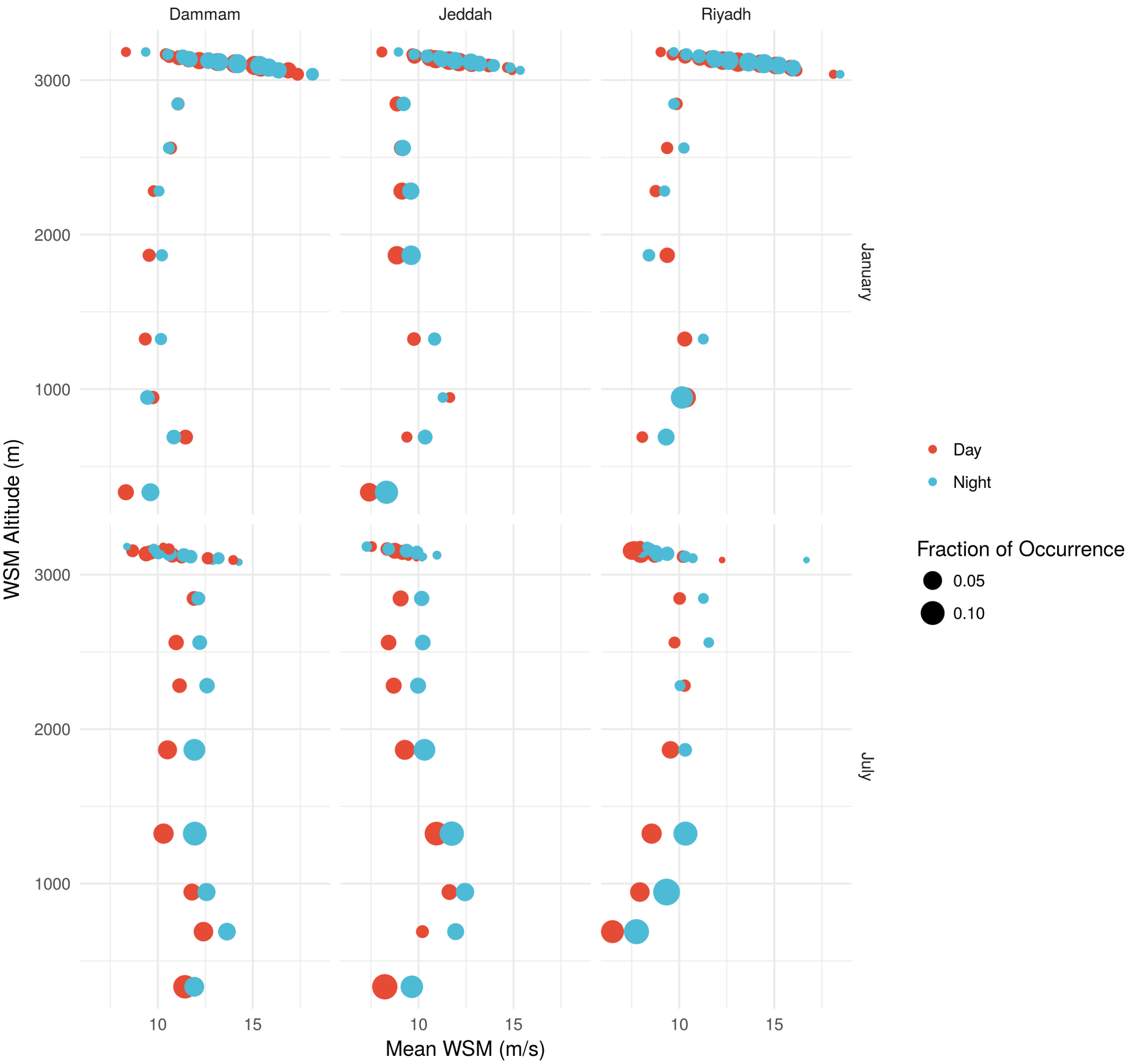

Figure S2: Wind speed maxima vertical profiles for 3 locations (Jeddah, Riyadh, Dammam) are illustrated, paneled by location and season. The altitudes of occurrence are clustered into 20 centers by k-means. The mean magnitudes of WSM are plotted for each altitude intervals for each location by season. The size of the points illustrates fraction of occurrences of maximum wind for each location by season.
